# Differential Responses of Fungal Community Diversity and Soil Environmental Variables to Freeze–Thaw Disturbance in Seasonally Frozen Soil

**DOI:** 10.3390/jof12030213

**Published:** 2026-03-16

**Authors:** Hong Pan, Xiaoyu Fu, Xiaosong Shan, Siyuan Liu, Dan Wei, Daoguang Zhu, Xinming Lu, Zhichao Cheng, Libin Yang

**Affiliations:** Key Laboratory of Biodiversity, Institute of Natural Resources and Ecology, Heilongjiang Academy of Sciences, Harbin 150040, China; panhong500@163.com (H.P.); 18646583130@163.com (X.F.); shanxiaosong2008@163.com (X.S.); liuliu9826@163.com (S.L.); weidan_0929@163.com (D.W.); zhudg01@163.com (D.Z.); luxinming0210@163.com (X.L.); chengzc928@163.com (Z.C.)

**Keywords:** cold-temperate, DNA sequencing, forest fungal response, permafrost, soil freeze–thaw cycle

## Abstract

Permafrost regions serve as sensitive indicators of global warming due to their ecological sensitivity and role as climate archives. To study how soil microbial communities in seasonal permafrost respond to freeze–thaw alternations, we analyzed composition and diversity during freezing, freeze–thaw, and thawing stages, identifying key taxa and environmental drivers. Our results identified 11 known fungal phyla and 13 dominant genera in permafrost regions. Most dominant fungi showed stable abundance during soil warming. However, the genera *Inocybe* and *Sebacina* were significantly suppressed when transitioning from frozen to freeze–thaw conditions. Fungal species diversity gradually increased with rising temperature and freeze–thaw frequency, with thawed soil showing higher richness and evenness. Frozen, freeze–thaw, and thawed soil were respectively associated with 90.48%, 71.43%, and 66.67% of node species. Adjacent stages shared 57.14% of coexisting species. Keystone node species declined progressively from frozen to thawed stages, indicating substantial yet continuous community reorganization. Furthermore, total carbon, organic carbon, available nitrogen, and phospholipid fatty acids peaked in freeze–thaw alternating soil. Active fungal biomass and species richness were most strongly correlated with soil carbon, temperature, and moisture. Overall, the influence of nutrients on soil fungi was limited across different freeze–thaw stages, while temperature emerged as the primary driver reshaping fungal community structure during freeze–thaw dynamics.

## 1. Introduction

Soil freeze–thaw is a cyclic process of repeated freezing and thawing occurring in the surface soil and subsoil layers. It is driven by diurnal or seasonal variations in thermal energy. This phenomenon is particularly prevalent in high-altitude and high-latitude permafrost regions [1]. With intensifying global climate change, soil freeze–thaw intensity and cycle frequency have undergone dramatic changes [2]. These changes profoundly impact soil microbial community structure and function, as well as the soil carbon budget of ecosystems.

Research on freeze–thaw effects has become a focal point in extreme environment studies. In existing research, how do freeze–thaw processes regulate the structure and function of soil microbial communities? Evidence indicates that soil freezing causes extensive microbial mortality via physical damage. Upon entering the freeze–thaw period, dead microbes release nutrients. Repeated freeze–thaw alternations disrupt soil aggregates [3]. Increased permeability enhances oxygen diffusion. These soil microenvironmental changes collectively enrich substrate availability and enhance the survival and activity of cold-tolerant microbes [4,5]. Other studies report that one freeze–thaw cycle can kill 50% of soil microbes and significantly reduce soil DNA by approximately 33% [6]. After multiple freeze–thaw alternations, soil microbial biomass and activity exhibit significant and widespread reductions [7,8,9]. However, microbial diversity often remains stable, suggesting adaptive resilience in alpine and polar soil [10]. Furthermore, meta-analyses reveal fungal communities’ higher responsiveness to freeze–thaw events than bacteria. Fungal diversity and co-occurrence network topology show pronounced shifts [11]. Given fungi’s higher carbon assimilation efficiency, freeze–thaw conditions can shift microbial dominance from bacteria to fungi [12,13]. This underscores the importance of studying fungal responses to heterogeneous soil environments under freeze–thaw stress.

In addition, how does freeze–thaw disturbance significantly influence the release and retention of soil carbon and nitrogen? It is generally believed that during freezing and thawing, microbial lysis releases intracellular organic matter, increasing soluble carbon and nitrogen in soil [14,15]. This process is considered detrimental to the storage of microbial biomass carbon and nitrogen [16]. Furthermore, ice crystal expansion and soil aggregate fragmentation also promote the mobilization of organic carbon and nitrogen and reduce their stability in permafrost [17]. In addition, evidence from other studies indicates that short-term freeze–thaw alternations can stimulate soil organic carbon mineralization, while long-term alternations tend to suppress mineralization rates. Moreover, increased freeze–thaw frequency and deeper freezing generally enhance nitrogen mineralization. This process promotes the production of NH_4_^+^-N and NO_3_^−^-N [18], but it also leads to an increase in nitrogen leaching [19]. Therefore, permafrost degradation has intensified the impacts of freeze–thaw action on soil carbon and nitrogen dynamics.

Our research team conducted a study on changes in the soil fungal network under freeze–thaw stress. The study was performed in situ in a large permanent fixed monitoring plot in a cold-temperate *Larix gmelinii* (Rupr.) Kuzen forest in China. This study sought to achieve three interrelated objectives: first, investigate the composition and diversity dynamics of soil fungal communities across freeze–thaw stages; second, examine how soil environmental factors vary under different thermal conditions; third, analyze associations between these soil environmental factors and key microbial taxa. The Greater Khingan Mountains were selected due to their sensitivity to climate change. Over the past 40 years, the warming climate has led to an increase in mean annual temperature of 0.30 °C per decade in the Greater Khingan Range [20]. This temperature rise has directly accelerated seasonal permafrost degradation and increased the thickness of the active layer. Furthermore, it has intensified microbial decomposition of organic matter, forming a “carbon bomb” effect. These characteristics make the region an ideal site for monitoring the ecological impacts of global warming.

## 2. Materials and Methods

### 2.1. Experimental Plot Overview

**Figure 1:**
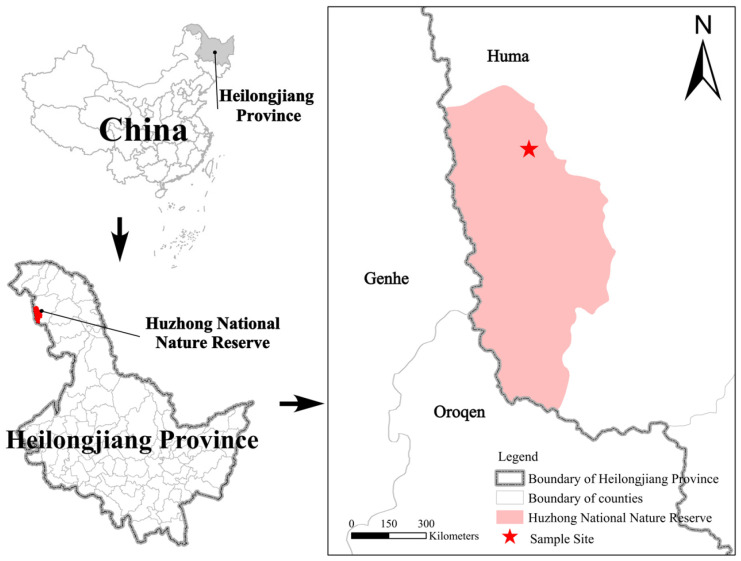
Map illustrating the geographical location of the research plot. The asterisk marks the sampling site in Heilongjiang Province, China. The map was created using QGIS 3.34 based on WGS 1984 coordinates, with administrative boundaries included.

As shown in Figure 1, the permafrost study area is located within the Huzhong National Nature Reserve in the Greater Khingan Range in China (51°49′01″–51°49′19″ N, 122°59′33″–123°00′03″ E). The experimental site lies within a 25 hm^2^ permanent forest dynamics plot established in a cold-temperate larch forest—part of the Global Forest Biodiversity Monitoring Network (CTFS-ForGEO). Within this large plot, three 1 hm^2^ satellite plots were selected for field experiments. The these satellite plots are situated on the northern slope of Yilehuli Mountain in the northern Greater Khingan Range, with an approximately 1000 m linear distance between adjacent plots.

All three plots are characterized by brown coniferous forest soil, gentle topography, and an average elevation of 910 m above sea level, with an elevation difference of less than 5 m across each plot. The region has a mean annual air temperature of −4 °C, relative humidity of 71%, and mean annual evaporation of 911 mm [21]. The vegetation is typical of boreal forests in northeastern China, with *L. gmelinii* as the dominant tree species, accompanied by co-dominant species including *Betula platyphylla*, *Pinus sylvestris* var. mongolica, and *Populus davidiana*. The understory shrub layer is primarily composed of *Spiraea* spp. and *Rosa* spp. Herbaceous composition varies slightly among the plots: in Plot 1, dominant herb species include *Deyeuxia*, *Artemisia*, *Galium*, *Lilium*, *Bupleurum*, and *Adenophora*; in Plot 2, the dominant herbs are *Artemisia*, *Galium*, *Polygonatum*, *Gentiana*, *Viola*, and *Filipendula*; and in Plot 3, they include *Deyeuxia*, *Galium*, *Lilium*, *Geranium*, *Filipendula*, and *Vicia*. This spatial arrangement ensures independence and replication across the three plots. Within each 1 hm^2^ plot, three 10 × 10 m subplots were established, spaced more than 100 m apart from one another, and each subplot contained three evenly distributed sampling points.

### 2.2. Soil Monitoring and Sample Collection

#### 2.2.1. Soil Temperature Monitoring

**Figure 2:**
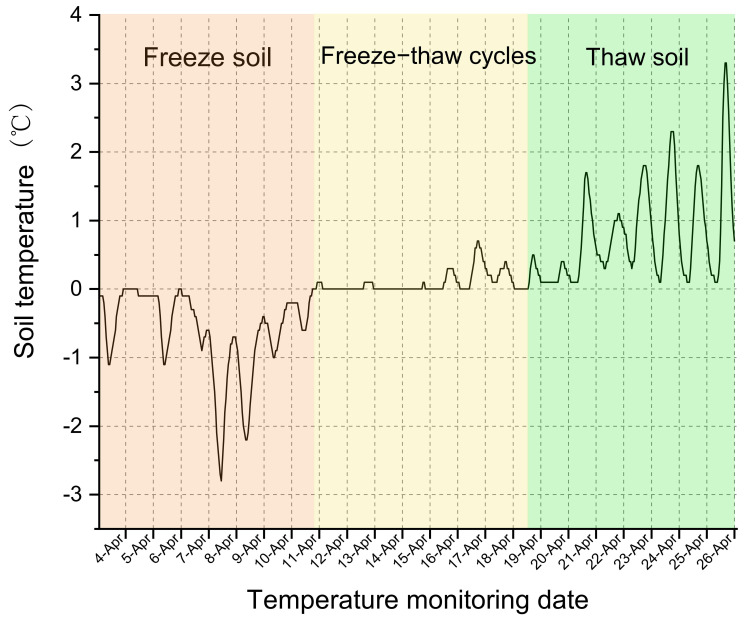
Schematic representation of the continuous soil temperature monitoring schedule, designed to accurately capture the onset and termination of soil freeze–thaw alternations.

During the soil freezing period (1 April 2024), an external temperature and humidity sensor (TM-THTR-4G, Tempconst, Xuzhou, China) was installed at a depth of 5–10 cm below the soil surface at each sampling point. The sensor had a temperature measurement range of −40 °C to 85 °C and a relative humidity detection range of 0–100% RH. As shown in Figure 2, remote, continuous monitoring of soil temperature and humidity was conducted to accurately track the initiation and termination of freeze–thaw events.

#### 2.2.2. Soil Sampling

To precisely capture the dynamics of freeze–thaw alternation (also known as a freeze–thaw cycle), a complete freeze–thaw alternation was defined as a transition in soil temperature from below 0 °C to above 0 °C. Based on data from the temperature sensors, the timing of the first freeze–thaw alternation was determined [22]. Soil samples were collected every two days starting from the initiation of the first alternation until the conclusion of the final alternation. In this experiment, FS (frozen soil) was collected prior to the first freeze–thaw alternation on 9 April 2024. Subsequently, FTS soil (freeze–thaw alternating soil) was collected on 12 April (the abbreviation of the sample name is FTS1), 14 April (FTS2), 16 April (FTS3), and 18 April (FTS4), and TS soil (thawed soil) was collected on 21 April following the completion of the last alternation.

The experimental design included six sampling days, with three plots established, each containing three subplots and nine sampling points per plot (three points per subplot). At each point, surface organic litter was removed, and approximately 200 g of soil was collected from the 0–20 cm depth. Samples were sealed, labeled, and transported to the laboratory. A total of 162 individual soil samples were collected.

Some of the collected soil samples were air-dried in a well-ventilated environment, during which plant and animal residues and stones were removed. The dried soil was then ground and passed through a 2 mm mesh sieve. From each sampling point, approximately 10 g of soil was subsampled. To minimize variability arising from spatial heterogeneity, composite samples were prepared by pooling the soil from nine sampling points within each plot on each sampling day. A total of 18 composite samples (each approximately 90 g) were obtained for the test of soil TC (total carbon), OC (organic carbon), TN (total nitrogen), and AN (available nitrogen). Each nutrient parameter was measured five times per composite sample, and the mean value was calculated after removing outliers.

The remaining portion of fresh soil was processed similarly: plant and animal residues and stones were manually removed, and the soil from nine sampling points within each plot was homogenized to form composite samples. A total of 18 fresh composite soil samples were prepared for fungal gene sequencing and the analysis of fungal PLFA (phospholipid fatty acid).

### 2.3. Extraction, Amplification and Library Construction of Fungal DNA

Soil microbial DNA was extracted using the E.Z.N.A.^®^ Soil DNA Kit (Omega Bio-tek, Norcross, GA, USA). The quality of the extracted genomic DNA was assessed by 1% agarose gel electrophoresis, and DNA concentration and purity were determined using the NanoDrop 2000 spectrophotometer (Thermo Fisher Scientific Inc., Waltham, MA, USA).

The fungal ITS gene region was amplified using the GeneAmp^®^ PCR System 9700 (Applied Biosystems Inc., Waltham, MA, USA) with barcoded primers ITS1F (5′-CTTGGTCATTTAGAGGAAGTAA-3′) and ITS2R (5′-GCTGCGTTCTTCATCGATGC-3′) [23]. The 20 μL reaction mixture consisted of 4 μL 5× FastPfu Buffer, 2 μL dNTPs (2.5 mM), 0.8 μL forward primer (5 μM), 0.8 μL reverse primer (5 μM), 10 ng template DNA, 0.4 μL FastPfu Polymerase, 0.2 μL BSA, and nuclease-free water added to a final volume of 20 μL.

Thermal cycling conditions were as follows: initial denaturation at 95 °C for 2 min; 25 cycles of 98 °C for 10 s, 55 °C for 30 s, and 72 °C for 90 s; followed by 24 cycles of 95 °C for 30 s, 60 °C for 30 s, and 72 °C for 45 s; a final extension at 72 °C for 2 min; and termination at 4 °C.

After PCR amplification, amplicons were separated via 2% agarose gel electrophoresis, and target bands were excised and purified using the PCR Clean-Up Kit (Major Yuhua Biomedical Technology Co., Ltd., Shanghai, China). Equal volumes of purified products with clear main bands and minimal contamination were pooled in a 1:1 ratio. DNA concentrations were quantified using the Qubit^®^ dsDNA HS Assay Kit and Qubit^®^ 4.0 Fluorometer (Thermo Fisher Scientific, USA). Only samples with the correct insert size and appropriate concentration were used for subsequent library construction. Purified PCR products were then subjected to library preparation using the NEXTflex^®^ Rapid DNA-Seq Kit (Bio Scientific, Avondale, AZ, USA) according to the manufacturer’s instructions.

### 2.4. High-Throughput Sequencing and Data Analysis

The PCR amplification products from 18 samples were sequenced using the Illumina Nextseq2000 platform (Illumina, San Diego, CA, USA). Following sequencing, raw paired-end reads were subjected to quality control using fastp v0.19.6 (https://github.com/OpenGene/fastp, accessed on 5 July 2025) and subsequently merged using FLASH v1.2.11 (https://github.com/dstreett/FLASH2, accessed on 7 July 2025) [24,25]. High-quality sequences were obtained after quality filtering and merging, and the sequence data were deposited into the NCBI Sequence Read Archive (SRA) under the accession number PRJNA1160036.

Effective sequences were clustered into operational taxonomic units (OTUs) at a 97% similarity threshold using UPARSE v7.0.1090 (http://drive5.com/uparse/, accessed on 8 July 2025) [26], with chimeric sequences removed during the process. Representative sequences for each OTU were obtained. Subsequently, the sequence data were normalized to the minimum sequencing depth across all samples. Alpha diversity indices were calculated using mothur v1.30.2 (https://mothur.org/wiki/calculators/, accessed on 8 July 2025) at the same 97% similarity level. Significant differences among diversity indices were assessed using the Kruskal–Wallis H test implemented in the stats package of R v3.3.1.

Taxonomic classification of OTU representative sequences was performed using the RDP classifier v2.11 (http://sourceforge.net/projects/rdp-classifier/, accessed on 9 July 2025) against the UNITE 9.0 ITS_fungi fungal taxonomy database, with a confidence threshold of 0.7. Species-level classification information was assigned at each taxonomic rank. Taxonomic abundance tables across different classification levels were generated using Qiime v1.91 (http://qiime.org/scripts/assign_taxonomy.html, accessed on 9 July 2025). The stats package in R v3.3.1 was further used to compare species distributions between two sample groups using Student’s *t*-test. *p*-values were adjusted for multiple comparisons using the false discovery rate (FDR) method. A two-tailed test was applied, and confidence intervals were calculated using Student’s t-distribution method at a 95% confidence level. Finally, the ggplot2 package in R v3.3.1 was employed to generate species distribution plots and bar charts showing significant differences at the phylum and genus levels.

A co-occurrence network analysis was conducted at the genus level using Networkx v1.11 to explore species coexistence patterns in environmental samples. Key species associated with soil freeze–thaw dynamics were identified based on network properties. The Mantel test was performed using the vegan v2.4.3 package in R to assess correlations between fungal community indices (PLFAs, Chao, and Simpson) and environmental variables across different freeze–thaw stages. The Bray–Curtis dissimilarity index was used as the distance metric, and Pearson correlation coefficients were visualized using heatmaps. Additionally, the pheatmap package in R was used to evaluate and visualize correlations between dominant fungal taxa and environmental factors, identifying key taxa influencing environmental variation.

### 2.5. Methods for Testing Soil Nutrients

TC (g/kg) was tested using combustion infrared absorption spectrometry with a Multi NC 2100S carbon–nitrogen analyzer (Multi NC 2100S, Analytik Jena, Jena, Germany). TN (g/kg) was measured using the Dumas combustion method with the same carbon–nitrogen analyzer. OC (g/kg) was quantified using the potassium dichromate volumetric method with external heating. AN (mg/kg) was determined via the alkali diffusion method [27].

### 2.6. Methods for Testing Soil Fungal PLFAs

PLFAs of fungi were detected to indirectly characterize the species composition and biomass of the living fungal community in the soil [28,29]. The detection of PLFAs is a supplement to the high-throughput sequencing, which cannot distinguish the DNA of living and dead microorganisms.

PLFAs were extracted and methylated using a potassium hydroxide–methanol solution, with nonadecanoic acid (C19:0) used as an internal standard [30,31]. The resulting fatty acid methyl esters were analyzed using an Agilent 6850 Series II Networked GC System (Agilent Technologies, Santa Clara, CA, USA). PLFA profiles were identified using the Sherlock Microbial Identification System (MIDI Sherlock, MIDI Inc., Newark, DE, USA).

### 2.7. Methods for Processing Statistical Data

Statistical analyses of soil environmental factors were conducted using IBM SPSS Statistics 19. Continuous soil temperature monitoring data were visualized using Origin v10.0.0.3920.

## 3. Results

### 3.1. Differences in Fungal Community Diversity Indices Across Different Soil Freeze–Thaw Stages

**Figure 3:**
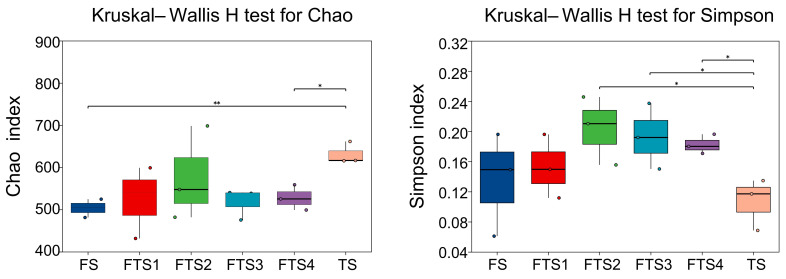
Boxplot illustrating alpha diversity indices of fungal communities based on OTUs during the soil freeze–thaw process. The abbreviations for the samples on the X-axis are defined as follows: FS denotes frozen soil, FTS1–4 denote the different batches of soil collected during the soil freeze–thaw alternation period, and TS denotes thawed soil. * indicates significant differences (*p* < 0.05). ** indicates extremely significant differences (*p* < 0.01) after Kruskal–Wallis H test.

After quality control, a total of 1,472,584 effective sequences were obtained, comprising 370,601,454 bases, with an average sequence length of 251 bp. Clustering yielded 1809 OTUs. The coverage values of fungal libraries for all samples exceeded 0.99, indicating sufficient sequencing depth; thus, the results of alpha diversity analysis reliably reflect the true composition of fungal communities in freeze–thaw soil. As shown in Figure 3, the Chao1 index was highest in TS, exhibiting significant (*p* = 0.0033) and highly significant (*p* = 0.0113) differences compared to FS and FTS4 soil, respectively. The Simpson index of TS was the lowest, showing statistically significant differences relative to FTS2 (*p* = 0.0457), FTS3 (*p* = 0.0460), and FTS4 (*p* = 0.0482) during the freeze–thaw alternating period. These findings suggest that soil fungal species diversity progressively increases with rising temperature and the frequency of freeze–thaw alternations. Frozen soil exhibited the lowest species richness (as indicated by the Chao1 index), whereas thawed soil demonstrated not only the highest species diversity (as reflected by the Simpson index) but also high levels of both richness and evenness, resulting in significant alpha diversity differences.

### 3.2. Composition and Distribution Differences of Fungal Communities Across Different Freeze–Thaw Stages of Soil

**Figure 4:**
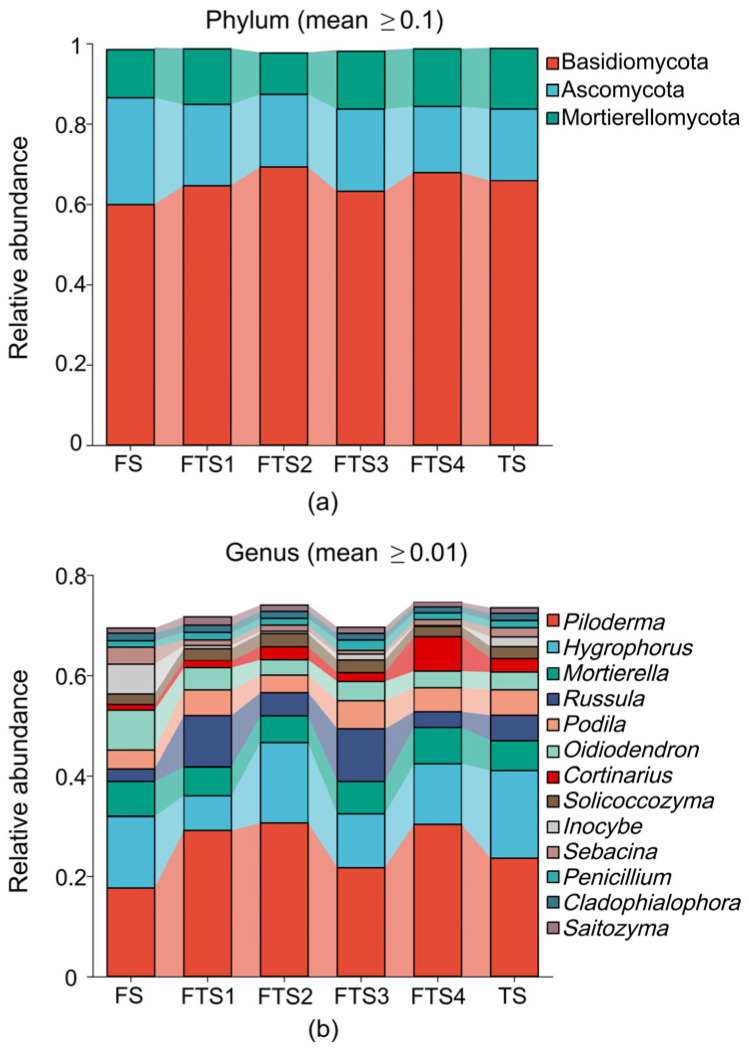
Composition of fungal communities in soil across different freeze–thaw stages at the (**a**) phylum and (**b**) genus levels.

As shown in Figure 4a, at the phylum level, a total of 11 known fungal phyla were identified in the permafrost region of the Greater Khingan Mountains during the freeze–thaw stages. Among these, Basidiomycota was the dominant phylum (mean relative abundance > 0.65), while Ascomycota and Mortierellomycota were relatively dominant phyla (0.13 < mean relative abundance < 0.20). The remaining phyla exhibited relative abundances below 0.01. No significant differences in phylum-level composition were observed across the different freeze–thaw stages.

**Figure 5:**
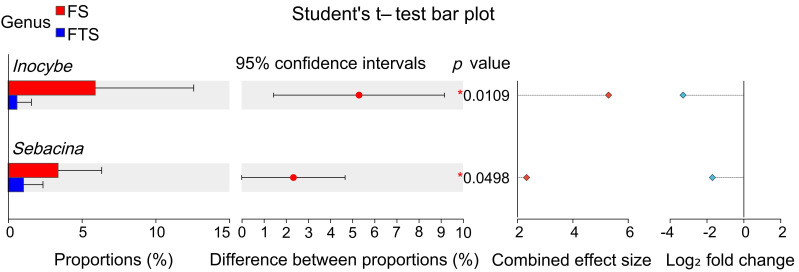
Statistical significance of fungal community differences between two soil freeze–thaw stages at the dominant genus level. There were no significant differences among the other unlisted groups. * indicates significant differences (*p* < 0.05) after Student’s *t*-test.

As shown in Figure 4b, at the genus level, 13 genera including *Piloderma, Hygrophorus*, *Mortierella*, *Russula*, *Podila*, *Oidiodendron*, *Cortinarius*, *Solicoccozyma*, *Inocybe*, and *Sebacina* were identified as dominant fungal taxa with relative abundances exceeding 0.01. Among them, *Piloderma* and *Hygrophorus* were classified as dominant genera (0.13 < mean relative abundance < 0.25), while the remaining genera were categorized as relatively dominant (0.01 < mean relative abundance < 0.06). T-test analysis (Figure 5) revealed that *Inocybe* (*p* = 0.0109) and *Sebacina* (*p* = 0.0498) exhibited significant differences between the frozen soil and freeze–thaw alternation soil. The abundance of both genera significantly decreased during the transition from frozen to freeze–thaw alternation conditions and did not recover until the soil entered a stable thawing stage, as indicated by the trend shown in Figure 4b. Supplementary Table S3 further revealed that although most dominant fungal genera did not show statistically significant differences in abundance across the freeze–thaw stages during soil warming, certain genera exhibited distinct dynamic patterns. For instance, *Mortierella* and *Oidiodendron* showed a gradual decline in abundance during the warming process, whereas *Piloderma*, *Russula*, and *Cortinarius* exhibited a transient increase followed by a decrease during freeze–thaw alternations. *Hygrophorus* and *Podila* declined during freezing but recovered during thawing. These findings suggest that the response of fungal communities to permafrost thawing is a complex process influenced by multiple environmental and ecological factors, warranting further investigation.

### 3.3. Coexistence Relationships of Fungal Species Across Different Freeze–Thaw Stages of Soil

**Figure 6:**
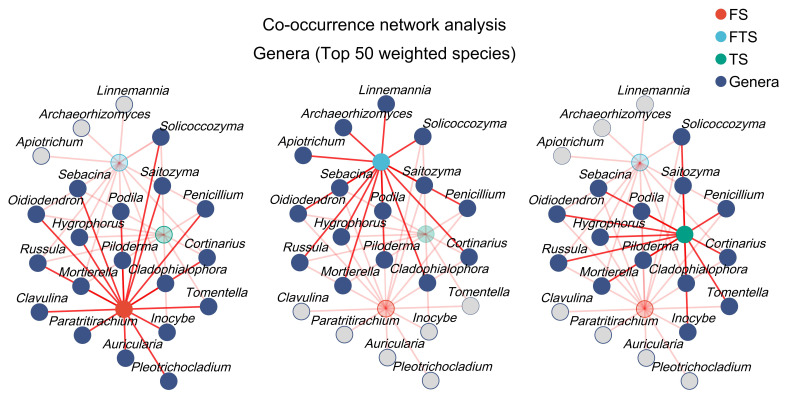
Co-occurrence network analysis revealing genus-level coexistence patterns across different soil freeze–thaw stages.

Co-occurrence network analysis (Figure 6) revealed that among the key node species with a weighted degree > 700, 90.48% were associated with the frozen soil system. As soil temperature fluctuated and increased, 71.43% of the species were linked to the freeze–thaw alternation stage, of which 57.14% coexisted with the frozen soil. Notably, *Linnemannia*, *Archaeorhizomyces*, and *Apiotrichum* were newly detected in freeze–thaw alternation soil, whereas *Clavulina*, *Paratritirachium*, *Auricularia*, and *Pleotrichocladium* disappeared. Upon complete thawing, 66.67% of the species were associated with the thawed soil system, with 57.14% coexisting with the freeze–thaw alternation soil. *Inocybe* and *Tomentella* emerged as newly detected genera in TS, while *Linnemannia*, *Archaeorhizomyces*, and *Apiotrichum* were no longer present. These results indicate a progressive decline in the number of key node species as the freeze–thaw alternation progresses. *Clavulina*, *Paratritirachium*, *Auricularia*, and *Pleotrichocladium* were exclusively detected in the frozen soil system and disappeared once soil temperatures exceeded the freezing point, suggesting that these species possess psychrophilic adaptations and may lose competitive advantages under non-frozen conditions. Similarly, *Linnemannia*, *Archaeorhizomyces*, and *Apiotrichum* were only observed during the freeze–thaw alternation stage. It is hypothesized that the transient availability of liquid water during freeze–thaw alternations may facilitate metabolic activation of these species, which appear to be more tolerant to osmotic stress induced by temperature fluctuations.

### 3.4. Soil Environmental Factors Across Different Freeze–Thaw Stages and Their Correlation with Fungal Communities

As shown in Table 1, the measured nutrients were lowest in frozen soil. TC, OC, and AN showed significant peaks (*p* < 0.05) in freeze–thaw alternation soil, compared with the frozen and thawed soil stages, and then significantly decreased after the soil was stably thawed. TN remained at a relatively high concentration from the initial stage of the freeze–thaw alternations until the thawed soil stage. Soil temperature and humidity monitoring data revealed that a small amount of unfrozen water existed in frozen soil below the freezing point. Soil moisture gradually increased with rising soil temperature and showed a significant stepwise increase across the three stages—frozen soil, freeze–thaw alternation soil, and thawed soil (*p* < 0.05). The initial fungal PLFA biomass was lowest in frozen soil and gradually increased with the rise in soil temperature. Compared with the frozen soil and thawed soil, PLFAs showed a significant peak (*p* < 0.05) during the mid-stage of freeze–thaw alternations (FTS2–FTS3), followed by a significant decline in the thawed soil stage (*p* < 0.05). These results indicate that soil fungal biomass responds strongly to the fluctuating freeze–thaw process.

**Figure 7:**
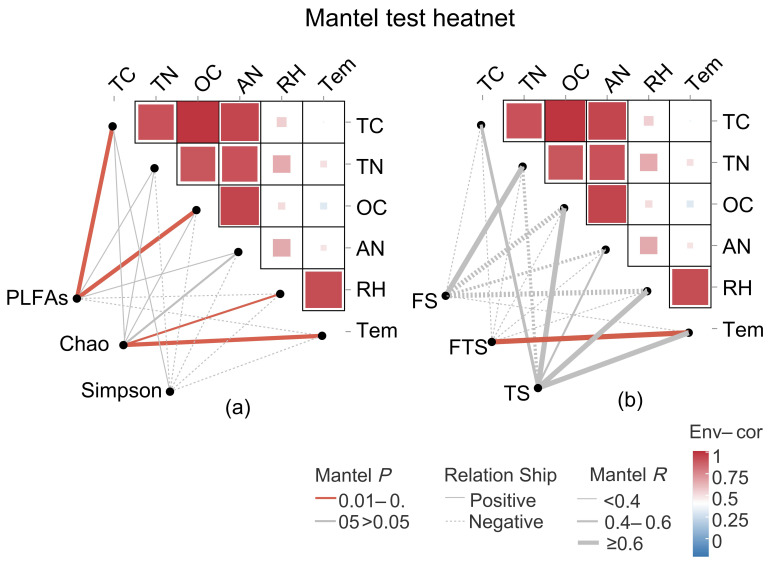
Mantel test analysis of the relationships between soil environmental factors and (**a**) fungal PLFAs and alpha diversity indices and (**b**) community composition across different soil freeze–thaw stages. In panel (**a**), the PLFAs and Chao and Simpson indices are all based on the data collected throughout the entire freeze–thaw period (from FS to FTS and then to TS).

Figure 7a presents the relationships between fungal PLFAs, Chao and Simpson indices, and soil environmental factors. PLFAs were significantly positively correlated with TC and OC, while Chao was significantly positively correlated with RH and Tem. These results indicate that carbon has the greatest influence on the biomass of active fungi during the freeze–thaw process and that fungal species richness (as indicated by the Chao index) responds most strongly to soil temperature and moisture. Figure 7b presents the correlations between microbial community structure at different freeze–thaw stages and soil environmental factors. Although FS showed a strong positive correlation with TN and TS showed strong positive correlations with TC, OC, AN, RH, and Tem to varying degrees, these correlations were not statistically significant. In contrast, the fungal community in freeze–thaw alternation soil showed a significant positive correlation with Tem. These results suggest that although soil carbon and nitrogen have some influence on fungal biomass (as indicated by PLFAs and Chao), overall, the impact of nutrients on soil fungi across different freeze–thaw stages is limited, and temperature is a key factor influencing the structure of fungal communities in freeze–thaw alternation soil.

**Figure 8:**
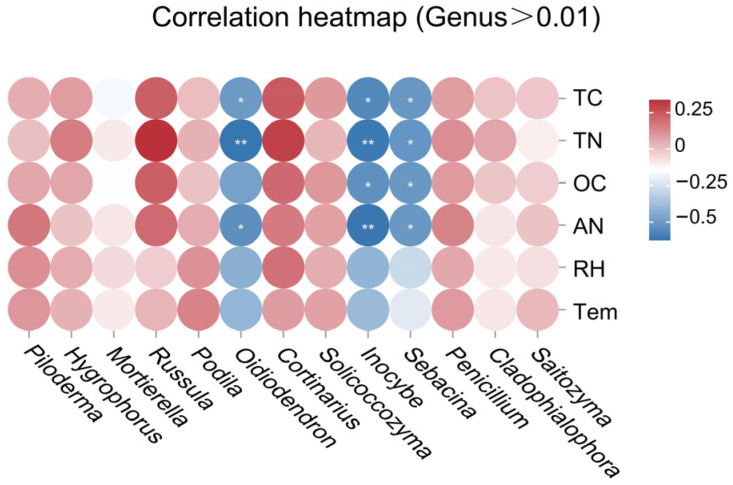
Heatmap analysis of the correlation between soil environmental factors and fungal genera during the soil freeze–thaw process. The data were collected throughout the entire freeze–thaw period (from FS to FTS and then to TS). * *p* < 0.05, significant correlation; ** *p* < 0.01, highly significant correlation.

From the perspective of correlations between fungal species and environmental factors (Figure 8), fungal species with relative abundances greater than 0.01 showed weak positive correlations with environmental factors (*r* < 0.35). In contrast, *Oidiodendron*, *Inocybe*, and *Sebacina* exhibited strong negative correlations with soil nutrients. We speculate that freeze–thaw alternations may cause most fungi to shift into a survival-based metabolic mode, thereby reducing the decomposition and utilization of soil nutrients. The negative correlation mechanisms between a few dominant fungal taxa and soil carbon and nitrogen require further verification through long-term field observation data.

## 4. Discussion

### 4.1. Seasonal Variations in Fungal Diversity Driven by Temperature During the Freeze–Thaw Process of Permafrost

Previous studies have shown that temperature is a primary driver of forest soil microbial communities [32]. Fungal communities exhibited sensitivity to temperature-driven freeze–thaw events [11]. In our study, thawed soil exhibited the highest fungal richness and evenness after the frozen period and freeze–thaw alternation stages. This aligned with previous findings that a complete thawing period significantly increased microbial community diversity [10]. This outcome stemmed from physical fragmentation induced by freeze–thaw alternations, which released more labile organic substrates [33]. It also resulted from the activation of dormant mesophilic fungal communities under warming conditions. Notably, significant differences in diversity indices were observed between the mid-to-late freeze–thaw alternations stages and the thawed stage. This suggested a threshold effect of freeze–thaw frequency on fungal survival. Repeated freeze–thaw alternations were survived by stress-tolerant species, whereas sensitive species declined or disappeared with increasing frequency, thereby reducing fungal diversity during the later stages of freeze–thaw alternations. This observation aligned with previous findings in alpine permafrost ecosystems that microbial communities under freeze–thaw alternations exhibited lower network complexity than those in fully thawed or frozen periods [34].

In our study, fungal diversity gradually increased with increasing frequency in freeze–thaw alternation soil. This finding aligned with the positive correlation between temperature/freeze–thaw frequency and diversity that was reported in high-altitude ecosystems. First, each thawing stage provided a metabolic window that enhanced microbial activity. A higher freeze–thaw frequency extended the cumulative duration of microbial activity, enabling more species to complete key life-cycle stages. Second, freeze–thaw alternations created spatiotemporal heterogeneity. An increase in initial frequency stimulated community diversity, whereas excessive frequency caused repeated community reorganization [35]. Third, rising temperatures and increasing freeze–thaw frequency expanded labile resources and reduced competitive exclusion. Fungal communities evolved adaptive strategies to synchronize their reproduction with freeze–thaw rhythms.

In the seasonal permafrost of the Greater Khingan Mountains, local fungal communities developed conserved adaptive mechanisms to cope with temperature fluctuations. An increase in moderate freeze–thaw frequency stimulated latent fungal diversity. This temperature-driven pattern of fungal diversity aligned with the theory that temperature regulated microbial diversity [36] and also provided counterevidence to the claim that warming reduces fungal richness [37].

### 4.2. Dynamic Responses of Community Composition and Species Co-Occurrence Relationships to Soil Freezing and Thawing Processes

In the Alps and the Tibetan Plateau permafrost ecosystems, researchers characterized the transcriptional responses of fungal communities before and after thawing. Ascomycota, Zygomycota, and Basidiomycota were the dominant phyla in permafrost [38], with Basidiomycota exhibiting significantly higher transcriptional activity during the thawing phase compared to the frozen state [34]. In this study, the dominant phyla detected—Basidiomycota, Ascomycota, and Mortierellomycota—showed no significant differences across freeze–thaw stages. This indicated their broad adaptability to the freeze–thaw process. Basidiomycota typically produced well-developed hyphae and formed symbiotic associations with local larch [39], which facilitated the acquisition of symbiotic resources under harsh environments. Basidiomycota tended to form sclerotia or chlamydospores, enabling resistance to freeze–thaw stress [13]. Moreover, Ascomycota acted as a more efficient decomposer in organic matter degradation [40], whereas Mortierellomycota exhibited stronger stress tolerance than other phyla [41]. The stability of these two phyla indicated that they had evolved adaptive strategies to cope with low-temperature fluctuations. Through dynamic adjustments of internal species composition, the dominant phyla maintained a strong community buffering capacity in the changing permafrost.

Previous studies have reported *Cladophialophora*, *Archaeorhizomyces*, *Pezoloma*, *Piloderma*, *Serendipita*, and *Hygrophorus* as dominant genera in alpine permafrost [38,42]. Of these, *Piloderma* and *Hygrophorus* were also identified as dominant taxa in our study of the Greater Khingan Mountains permafrost. We attributed their dominance primarily to mycorrhizal associations with native tree species [43,44]. This mutualistic symbiosis provided a stable carbon source for fungi [45,46]. Furthermore, *Inocybe* and *Sebacina* have also been reported to be widely present in the soil of arctic-alpine tundra, alpine meadows, and subalpine forests [47,48]. In our study, the abundance of these two genera decreased significantly after soil shifted from frozen to freeze–thaw alternations and recovered only after complete soil thawing. Such a V-shaped response indicated that these genera were selectively inhibited under temperature fluctuation stress, and population recovery was promoted only when temperatures stabilized during the thawing period.

Fungal co-occurrence network analysis revealed the patterns of species coexistence and differentiation. First, 90.48% of node taxa were associated with frozen soil. This high proportion indicated that the species filtered by frozen soil exhibited cold tolerance. Reduced metabolic activity weakened interspecific competition, leading to a high proportion of species coexistence [49]. Second, 71.43% of taxa were associated with freeze–thaw alternation soil, and 57.14% co-occurred with those in frozen soil. The directional turnover of newly emerged taxa, as well as disappeared taxa, reflected the unique environmental selection pressure during the freeze–thaw alternation stage. Third, 66.67% of taxa were associated with thawed soil, and 57.14% co-occurred with those in freeze–thaw alternation soil. The decreased proportion of coexisting taxa suggested that although many fungi survived the physical pressure of freeze–thaw alternations, they failed to persist in thawed soil where nutrient competition intensified. Collectively, despite high shared taxa proportions across soil freeze–thaw stages, temperature-driven species turnover revealed the complex responses of fungi to permafrost changes [21]. Fungal communities underwent significant species restructuring while maintaining a certain degree of species continuity.

### 4.3. Correlation Analysis of Soil Nutrients and Fungal Communities Triggered by Freeze–Thaw Alternations

Compared with the freezing and thawing phases, fungal communities exhibited a significant positive correlation with temperature only during the freeze–thaw alternation phase, highlighting temperature’s critical filtering role in community reassembly. Dominant fungal species (relative abundance > 0.01) showed weak positive correlations with environmental factors, indicating the strong adaptability of these widespread taxa to freeze–thaw processes. Conversely, low-abundance genera, including *Oidiodendron, Inocybe,* and *Sebacina*, were strongly negatively correlated with soil nutrients, suggesting their response strategies to freeze–thaw stress were most likely linked to oligotrophy and competitive exclusion [50]. These findings extended the application of niche theory to permafrost ecosystems, via the disassembly and reassembly of microbial communities during freeze–thaw alternation. Furthermore, the significant positive correlation between temperature and Chao1 confirmed that soil temperature was a key driver of fungal species diversity in freeze–thaw soils.

Low temperatures reduced microbial activity and inhibited the decomposition of organic matter, resulting in the lowest soil C and N contents during the freezing period. This was consistent with the widely reported conclusion that low temperatures suppressed organic matter mineralization [36,51]. In this study, soil OC, TC, AN and TN exhibited significant peaks in soils during the freeze–thaw alternation period (*p* < 0.05). We attributed this result primarily to the freeze–thaw priming effect mechanism. Firstly, repeated temperature fluctuations accelerated microbial lysis, thereby releasing more organic substances [16]. Secondly, the breakdown of soil aggregates during freeze–thaw processes exposed additional C and N pools [17]. In this study, our results further demonstrated that OC and TC began to decline before the completion of the freeze–thaw alternations, whereas AN and TN decreased only after the soil thawed stably; during the same freeze–thaw period, C and N decreased by 12.8% and 0.04%, respectively. This finding indicated that C was more sensitive to the duration of freeze–thaw alternations than N [52]: short-term freeze–thaw alternations exerted a priming effect on C, while long-term alternations inhibited the rate of C mineralization. In contrast, N could maintain high concentrations for an extended period during freeze–thaw alternations, primarily because N is an essential substrate for sustaining microbial activity: only continuous N mineralization could support the repeated reconstruction of microbial communities under freeze–thaw stress. However, the effects of freeze–thaw processes on N mineralization varied [53], and evidence for N accumulation and loss induced by freeze–thaw treatments has remained scarce to date.

Furthermore, PLFAs exhibited a low-high-low phasic response during the soil freezing → freeze–thaw cycle → thawing process, a pattern consistent with the variations in soil C and N. This indicated that the PLFA peak observed during freeze–thaw alternations was also linked to the freeze–thaw priming effect. Although freeze–thaw alternations caused the death of some microorganisms, they also provided favorable conditions for the succession and proliferation of surviving microbial communities [54]. Additionally, studies have shown that nutrient substrates released by short-term thawing of permafrost during freeze–thaw alternations were the primary drivers of shifts in soil microbial community structure [55]. In our study, the strong correlation between PLFAs, TC and OC demonstrated a close link between microbial activity and C dynamics. This implied that the freeze–thaw alternation period constituted a critical window for soil C release and that substrate C was a key factor regulating the biomass of active fungi.

## 5. Conclusions

Dominant fungal taxa observed in the seasonal permafrost showed no significant variation in relative abundance across different freeze–thaw stages, suggesting strong buffering capacity and broad adaptability. However, genera like *Inocybe* and *Sebacina* had reduced abundance during the transition from frozen to freeze–thaw conditions.

Co-occurrence network analysis indicated a gradual decline in keystone species with advancing freeze–thaw progression, yet a high proportion of species co-occurred across stages, reflecting community continuity despite reorganization.

Fungal diversity remained stable during freeze–thaw alternations, while thawed soil at higher temperatures showed increased richness and evenness.

Nutrient association analysis revealed that many fungi shifted toward survival-maintaining metabolic strategies. Most nutrients had limited influence on fungal composition. Substrate carbon significantly affected active fungal biomass, and temperature emerged as a key driver altering community structure in freeze–thaw soil.

In short, these findings confirm the freeze–thaw process as an “ecological engineer” that both creates new niches through physical disturbance and reshapes community structure via selective pressures.

## Figures and Tables

**Table 1:** Soil environmental factors across different freeze–thaw stages. The abbreviations are defined as follows: TC denotes total carbon, TN denotes total nitrogen, OC denotes organic carbon, AN denotes available nitrogen, RH denotes relative humidity, Tem denotes soil temperature, and SD denotes standard deviation of the statistical data. Values within the same column followed by the same lowercase letter indicate no significant difference, while those with different lowercase letters indicate significant differences (*p* < 0.05).

Sample	Soil Environmental Factors (Mean ± SD)						
	TC (g/kg)	TN (g/kg)	OC (g/kg)	AN (mg/kg)	RH (%)	Tem (℃)	PLFAs (nmol/g)
FS	44.37 ± 1.62 d	1.72 ± 0.14 c	36.42 ± 0.22 e	0.17 ± 0.01 c	5.77 ± 0.06 e	−1.10 ± 0.20 d	71.26 ± 5.55 e
FTS1	78.45 ± 1.98 c	2.17 ± 0.12 b	69.39 ± 2.89 c	0.24 ± 0.00 b	10.70 ± 0.00 d	0.00 ± 0.00 c	99.86 ± 7.28 c
FTS2	100.45 ± 7.70 a	2.42 ± 0.10 a	96.38 ± 5.22 a	0.28 ± 0.01 a	15.57 ± 0.15 c	0.00 ± 0.00 c	138.24 ± 6.19 a
FTS3	99.71 ± 1.50 a	2.48 ± 0.20 a	93.18 ± 2.35 a	0.28 ± 0.01 a	15.50 ± 0.10 c	0.27 ± 0.06 b	130.64 ± 7.92 a
FTS4	86.93 ± 6.38 b	2.40 ± 0.06 ab	81.40 ± 3.66 b	0.27 ± 0.01 a	18.37 ± 0.15 b	0.30 ± 0.00 b	109.90 ± 5.23 b
TS	73.69 ± 3.39 c	2.24 ± 0.17 ab	63.01 ± 4.74 d	0.24 ± 0.01 b	22.20 ± 0.50 a	1.67 ± 0.06 a	87.90 ± 5.97 d

## Data Availability

The data presented in this study are openly available in NCBI at https://dataview.ncbi.nlm.nih.gov/object/PRJNA1160036, reference number PRJNA1160036.

## Supplementary Materials

The following supporting information can be downloaded at: https://www.mdpi.com/article/10.3390/jof12030213/s1, Table S1. Statistical analysis of optimized sequence information after quality control. Table S2. Diversity index and significance test for inter-group differences. Table S3. T-test for the soil fungal bundance differences between the two groups during the three freeze-thaw stages.
