# Supplementary material for: Differential Responses of Fungal Community Diversity and Soil Environmental Variables to Freeze–Thaw Disturbance in Seasonally Frozen Soil
Source: J Fungi (Basel). 2026 Mar 16;12(3):213. doi: 10.3390/jof12030213 (PMC13028596; doi:10.3390/jof12030213)
Supplement: Supplementary file 1 [file jof-12-00213-s001.zip › Legends for Supplementary Materials.pdf]

Legends for Supplementary Materials

Table S1. Statistical analysis of optimized sequence information after quality control

Table S2. Diversity index and significance test for inter-group differences

Table S3. T-test for the soil fungal bundance differences between the two groups during the three freeze-thaw stages
